# SLC10A7 regulates O-GalNAc glycosylation and Ca^2+^ homeostasis in the secretory pathway: insights into SLC10A7-CDG

**DOI:** 10.1007/s00018-024-05551-2

**Published:** 2025-01-08

**Authors:** Zoé Durin, Aurore Layotte, Willy Morelle, Marine Houdou, Antoine Folcher, Dominique Legrand, Dirk Lefeber, Natalia Prevarskaya, Julia Von Blume, Valérie Cormier-Daire, François Foulquier

**Affiliations:** 1 Univ. Lille, CNRS, UMR 8576 – UGSF - Unité de Glycobiologie Structurale Et Fonctionnelle, 59000 Lille, France; 2 Inserm U1003, Laboratory of Excellence, Ion Channels Science and Therapeutics, Equipe Labellisée Par La Ligue Nationale Contre Le Cancer, GIS ONCO Lille, University of Lille, Lille, France; 3 Department of Neurology, Donders Institute for Brain, Cognition and Behavior, Radboud University Medical Center, 6525 GA Nijmegen, The Netherlands; 4 Translational Metabolic Laboratory, Department of Laboratory Medicine, Radboud Institute for Molecular Life Sciences, Radboud University Medical Center, 6525 GA Nijmegen, The Netherlands; 5 Department of Cell Biology, Yale University School of Medicine, New Haven, CT USA; 6 INSERM UMR1163, Institut Imagine, Université de Paris, Paris, France; 7 Service de Génétique Clinique, Centre de Référence Pour Les Maladies Osseuses Constitutionnelles, AP-HP, Hôpital Necker-Enfants Malades, Paris, France

**Keywords:** Glycosylation, SLC10A7, CDG, O-GalNAc

## Abstract

Glycans are known to be fundamental for many cellular and physiological functions. Congenital disorders of glycosylation (CDG) currently encompassing over 160 subtypes, are characterized by glycan synthesis and/or processing defects. Despite the increasing number of CDG patients, therapeutic options remain very limited as our knowledge on glycan synthesis is fragmented. The emergence of CDG resulting from defects in ER/ Golgi homeostasis makes this even more difficult. SLC10A7 belongs to the SLC10 protein family, known as bile acid and steroid transport family, exhibiting a unique structure. It shows a ubiquitous expression and is linked to negative calcium regulation in cells. The mechanisms by which SLC10A7 deficiency leads to Golgi glycosylation abnormalities are unknown. The present study identifies major *O*-glycosylation defects in both SLC10A7 KO HAP1 cells and SLC10A7-CDG patient fibroblasts and reveals an increased ER and Golgi calcium contents. We also show that the abundance of COSMC and C1GALT1 is altered in SLC10A7-CDG patient cells, as well as the subcellular Golgi localization of the Ca^2+^-binding Cab45 protein. Finally, we demonstrate that supraphysiological manganese supplementation suppresses the deficient electrophoretic mobility of TGN46 by an aberrant transfer of GalNAc residues, and reveal COSMC Mn^2+^ sensitivity. These findings provide novel insights into the mechanisms of Golgi glycosylation defects in SLC10A7-deficient cells. They show that SLC10A7 is a key Golgi transmembrane protein maintaining the tight regulation of Ca^2+^ homeostasis in the ER and Golgi compartments, both essential for glycosylation.

**Supplementary Information:**

The online version contains supplementary material available at 10.1007/s00018-024-05551-2.

## Introduction

Congenital disorders of glycosylation (CDG) are a group of rare genetic defects in glycosylation first described clinically in the 1980s. This currently encompasses over 160 CDG, most of them due to variants in genes involved in glycan synthesis, processing and attachments [1, 2]. In particular, defects in regulatory actors of glycosylation, particularly affecting the Golgi apparatus (such as vesicular trafficking, metal and pH homeostasis) constitute an emerging group of CDG [3, 4].

SLC10A7-CDG was first reported in 2018 and shows mainly skeletal dysplasia, amelogenesis imperfecta and decreased bone mineral density [5, 6]. SLC10A7 belongs to the SLC10 protein family which includes transporters primarily involved in bile acids, sulfated hormones and steroid transport [7–9]. Unlike other SLC10 family members, SLC10A7 has an atypical structure with 10 transmembrane domains and is widely expressed [10, 11]. Karakus and collaborators established SLC10A7 as a novel negative regulator of intracellular calcium signaling that most likely acts via STIM1, Orai1 and/or SERCA2 inhibition. Its exact cellular role remains undetermined [12, 13]. The involvement of SLC10A7 in cellular Ca^2+^ homeostasis fits with the human and mice phenotypes presenting enamel and bones anomalies, growth plate disorganization and decreased calcium deposits in bone mineralization in *Slc10a7*-deficient zebrafish. As for TMEM165 deficiency that affects Golgi manganese homeostasis, the glycomic signature is unique with an overall impairment of the Golgi glycosylation process characterized by strong *N*- and *O*-glycosylation defects, as well as a decrease in heparan sulfate production [5, 6]. SLC10A7 therefore appears as an essential regulator of Golgi glycosylation but the molecular mechanism by which a lack of SLC10A7 affects Golgi glycosylation remains unknown.

The aim of this study is to understand how a lack of SLC10A7 could affect Golgi glycosylation. We used SLC10A7-deficient CDG fibroblast and isogenic SLC10A7 KO HAP1 cells and present evidence that the major *O*-glycosylation defects observed in SLC10A7 deficiency likely result from defective Ca^2+^ homeostasis in the secretory pathway. The lack of SLC10A7 leads to a strong decrease in both COSMC and C1GALT1 proteins as well as a complete absence of Golgi localization of the Ca^2+^-binding Cab45 protein.

## Material and methods

### Cell culture and treatments

HAP1 (Wild-type and SLC10A7 KO cells) were obtained from Horizon Discovery (HZGH005271c011). Patient fibroblasts from P1 and P2 were obtained from Pr Dirk Lefeber, Radboud University, The Netherlands, and patient fibroblasts P3, P4, and P5 from Pr Valérie Cormier-Daire, Necker Hospital, Paris, France. HAP1 cells were harvested in IMDM and fibroblasts in DMEM (Lonza, Basel, Switzerland), supplemented with 10% Fetal Bovine Serum (FBS) (Pan, Pan Biotech), in a humidified atmosphere with 5% CO_2_ at 37 °C. For drug treatment, cells were incubated with MnCl_2_ or Ac_3_Bn-α-GalNAc (Sigma-Aldrich, St Louis, Missouri, USA) for different times/ concentrations as indicated in the figures and their legends.

### Western blot

For cell lysate, cells were scrapped on ice, in PBS (Euromedex, Souffelweyersheim, France), placed in Eppendorf tubes and centrifugated for 10 min at 4 °C. The supernatants were eliminated, and cell pellets were lysed by 20 aspirations/discharges with RIPA buffer [Tris/HCl 50 mM pH 7.9, NaCl 120 mM, NP40 0.5%, EDTA 1 mM, Na_3_VO_4_ 1 mM, NaF 5 mM] supplemented with a protease cocktail inhibitor (Roche Diagnostics, Penzberg, Germany), and vortexed twice for 10 s. Samples were then centrifugated for 30 min at 4 °C, 20,000 g. Protein concentration contained in the supernatant was estimated with the micro-BCA Protein Assay Kit (Thermo Scientific). For proteins coming from cell culture media, proteins were precipitated by adding a methanol volume corresponding at 3 times the cell culture media volume, and 0.75 volume of chloroform. After vortexing for 15 s, 2 volumes of water were added and tubes were centrifugated for 15 min at 4 °C, 869 g. The supernatant above proteins was discarded, and the sample was mixed with 2.25 volumes of methanol and centrifugated in the same conditions. The whole supernatant was discarded, and the protein pellet was resuspended in RIPA, and transferred to a microtube, before 30 min of centrifugation at 20,000 g, 4 °C. Fifteen µL of the supernatant were mixed with loading NUPAGE blue on a gel. Western blot samples of cells were prepared by mixing 10 (LAMP2, TGN46) or 20 µg of protein lysate (C1GALT1, COSMC) with water to reach 15 µL, and 5 µL of NuPAGE LDS (Invitrogen Carlsbad, California) supplemented with 16% of beta-mercaptoethanol were added. Samples were heated for 10 min at 95 °C (except for COSMC), separated on 4–12% BisTris gels (Invitrogen, Carlsbad, California), and then transferred with the iBlot 2 system (Program 0–7 min, Invitrogen, Carlsbad, California). Membranes were blocked for 1–3 h in TBS-Tween-0.05% + 5% dry milk or BSA (fraction V, Roche Diagnostics, Penzberg, Germany). Primary antibodies (see antibodies table) were incubated overnight in the blocking solution, at 4 °C under agitation. The day after, membranes were washed three times with TBS-Tween, and secondary antibodies were diluted in the blocking solution and incubated for 1 h at room temperature. Membranes were washed five times for 5 min with TBS-Tween. Signal was detected with a chemiluminescence reagent (ECL 2 Western Blotting Substrate or SuperSignal West Pico PLUS chemiluminescent Substrate, Thermo Scientific) in a dark room on imaging film (GE Healthcare, Little Chalfont, United Kingdom).

### Immunofluorescence

Cells harvested on glass coverslips were fixed with 4% paraformaldehyde for 20 min, washed 3 times and coverslips were mounted in a wet chamber. Cells were permeabilized with PBS + 0.5% Triton-X100 for 10 min and washed with PBS three times, then blocked for 1 h in blocking buffer [0.2% gelatin, 2% Bovine Serum Albumin (BSA), 2% Fetal Bovine Serum (FBS) (Lonza) in PBS]. Primary antibodies were diluted at 1/100 in blocking buffer and incubated for 1 h. After 3 washes with PBS, secondary antibodies (GAR-Alexa Fluor 568 or GAM-Alexa fluor 488) were incubated for 1 h at 1/600. Cells were washed 3 times with PBS, and nucleus were stained for 10 min with 5 µg/mL DAPI blue. Cells were then mounted with 6 µL of Mowiol on glass slides after 10 rinses with deionized water. Fluorescence was detected through an inverted Zeiss LSM780 or LSM700 confocal microscope. Acquisitions were done using the ZEN pro 2.1 software (Zeiss, Oberkochen, Germany), and images were treated with ImageJ (Fiji).

### Lectin staining

For fluorescent lectin: coverslips were fixed and mounted in a wet chamber as described in the previous section. Paraformaldehyde was neutralized with 50 µM NH_4_Cl for 15 min, and coverslips were washed three times. Permeabilization, if needed, was performed with a mix of PBS + 0.1% BSA + 0.075% saponin, incubated for 15 min. Lectin *(Vicia Villosa Lectin* (VVL) recognized the O-GalNAc (= Tn Antigen) coupled to Fluorescein-Vector Laboratories, Newark, California, United States) were then incubated at 1/1000 in PBS + 0.1% BSA for 1 h, protected from light. After 3 washes, DAPI, mounting, and image acquisition were performed as described in the “immunofluorescence” section. For lectin-blot, samples preparation and migration were conducted as described in the Western blot section. Proteins were transferred on nitrocellulose membrane with the liquid Invitrogen (Carlsbad, California) transfer system, for 1 h at 10 V. Membranes were blocked 1 h in filtered 2% PVP—0.05% TBS-Tween. Biotinylated lectins (Vector Laboratories, Newark, California, United States) were incubated at 2 µg/mL in the same buffer for 45 min, and then washed 3 times for 15 min with TBST. Antibiotin-HRP antibodies (Sigma Aldrich, St-Louis, Missouri, United States) were diluted in the blocking buffer at 1/10000, incubated for 1 h and washed 6 times for 15 min with TBST. Signal was detected with chemiluminescence reagent (ECL 2 Western Blotting Substrate or SuperSignal West Pico PLUS chemiluminescent substrate, Thermo Scientific) using a CCD camera (FUSION Solo, Vilber Lourmat, France) and the FUSION-Capt Solo software for acquisition.

### Calcium imaging analysis

Patient fibroblasts were grown on glass coverslips to carry out cell imaging experiments. Ratiometric dye Fura-2/AM (F1221, Invitrogen) was used as a Ca^2+^ indicator. Cells were loaded with 2 μM Fura-2/AM for 45 min at 37 °C and 5% CO_2_ in corresponding medium and subsequently washed three times with external solution containing (in mM): 140 NaCl, 5 KCl, 1 MgCl_2_, 2 CaCl_2_, 5 Glucose and 10 Hepes (pH 7.4). The coverslip was then transferred in a perfusion chamber on the stage of Nikon Eclipse Ti microscope. Fluorescence was alternatively excited with a monochromator (Polychrome IV, TILL Photonics Gmbh) at 340 and 380 nm (for Ca-imaging experiments). Then, fluorescence was captured at 510 nm by a QImaging CCD camera (QImaging). Acquisition and analysis were performed with MetaFluor 7.7.5.0 software (Molecular Devices Corp.).

### Benzyl-GalNAc mass spectrometry analysis

The protocol used for Bn-GalNAc glycosylation status analysis by mass spectrometry (MALDI-QIT-TOF, Matrix-Assisted Laser Desorption/Ionisation-Quadrupole Ion Trap Time-Of-Flight) was described in Durin, Houdou, 2022 [14]. The same protocol was used, except for HAP1 cells that were cultured in IMDM complemented with 5% FBS, instead of DMEM.

### Deglycosylation assay

The deglycosylation assay was performed with the Agilent Enzymatic Deglycosylation Kit for N-Linked and Simple O-Linked Glycans (GK80110) and Agilent Extender Kit for Complex O-Linked Glycans (GK80115), according to manufacturer’s instructions (Agilent, Santa Clara, CA 95051 United States). Briefly, cells were lysed in RIPA buffer as described above, without protease inhibitors, in order to reach a concentration of 100 µg of protein in a maximum volume of 30 µL. Fifty to 100 µg of protein were heated at 100 °C for 10 min in 10 µL of incubation buffer supplemented with 2.5 µL of denaturation buffer and cooled down on ice. 2.5 µL of detergent solution and 1 µL of required enzymes were added, and samples were incubated for at least 3 h at 37 °C under agitation. The adequate volume to reach 10 µg of proteins per sample was then submitted to the Western blot protocol detailed above.

**Table:** 

	Antibody (specie)	Gel and samples preparation	Buffer	Dilution primary	Dilution secondary
Western blot	TGN46-Biorad (Sheep) #AHP500G	Precast – 10 µg-Heated	Milk 5%	1/1000 Overnight	Donkey anti-Sheep (Daiko) 1/10000
	LAMP2-Santa Cruz (Mouse) #sc-18822	Precast – 10 µg-Heated	Milk 5%	1/2000 Overnight	Goat anti-Mouse (Daiko) 1/20000
	COSMC–Santa Cruz (Mouse) #sc-271829	Homecast (20 µg)-Heated	BSA 5%	1/500 Overnight	Goat anti-Mouse (Daiko) 1/20000
	C1GALT1-Santa Cruz (Mouse) #sc-100745	Precast-20 µg-Heated	Milk 5%	1/1000 Overnight	Goat anti-Mouse (Daiko) 1/20000
	β-Actin – Sigma Aldrich (Mouse) #A1978	Homecast or Precast-10 to 20 µg-Heated or not	Milk or BSA 5%	1/10000 Overnight or 1 h	Goat anti-Mouse (Daiko) 1/20000
	Cab45-Sigma Prestige (Rabbit) #HAP011249	Homecast-10 µg-Not heated	BSA 5%	1/1000 Overnight	Goat anti-Rabbit (Daiko) 1/5000

**Table:** 

	Antibody (specie)	Dilution primary	Dilution secondary (Alexa Fluor)
Immunofluorescence	GM130 – BD Bioscience (mouse) #610,822	1/100	GAM488 1/600
	Giantin – Biolegend Covance (rabbit) #909,701	1/100	GAR568 1/600
	Cab45 – Sigma Prestige (Rabbit) #HAP011249	1/100	GAR568 1/600

## Results

### Loss of SLC10A7 leads to severe Golgi O-GalNAc glycosylation defects in SLC10A7-CDG patient fibroblasts and isogenic SLC10A7 KO HAP1 cells that are suppressed by high MnCl_2_ concentrations

**Fig. 1:**
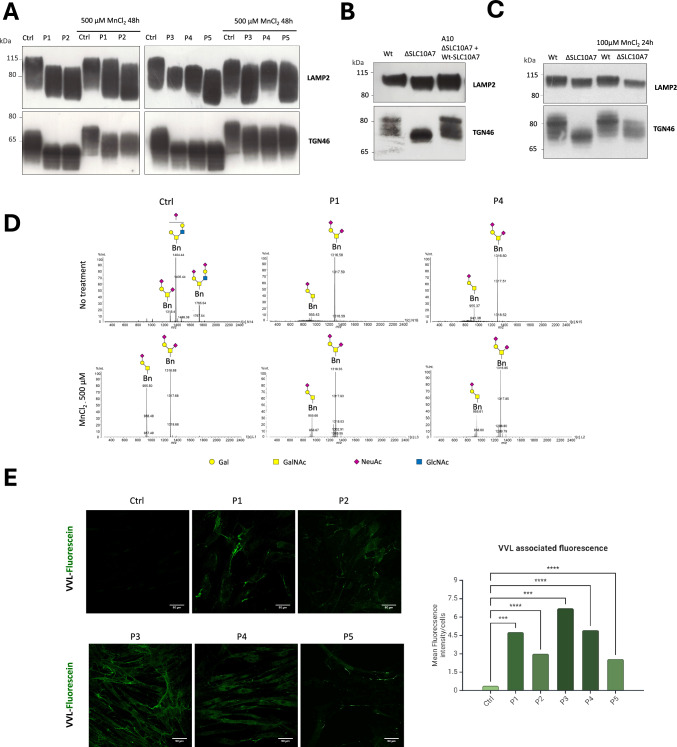
SLC10A7 KO cells and SLC10A7-CDG patient cells exhibit a strong *O*-glycosylation defect. **A** Western blot profiles of LAMP2 and TGN46 in five SLC10A7-CDG patient cells, treated or not with 500 µM MnCl_2_ for 48 h. **B** Western blot profiles of TGN46 and LAMP2 in isogenic Wt, SLC10A7 KO or complemented A10 HAP1 (SLC10A7 KO + Wt-SLC10A7) cells. **C** TGN46 and LAMP2 Western blot migration profiles of Wt or SLC10A7 KO HAP1 cells, treated or not with 100 µM MnCl_2_. **D** MALDI-QIT-TOF spectra of peracetylated Benzyl-GalNAc (Bn-GalNAc) glycosylation status in control, and patient 3 and 4 fibroblasts, treated with 50 µM Ac_3_-Bn-GalNAc, with or without 500 µM MnCl_2_ treatment for 72 h. The symbols depicting the different monosaccharides are found at the bottom of the figure. **E** Confocal images of non-permeabilized control and patient fibroblasts stained with *Vicia villosa* lectin (VVL) – fluorescein and their quantification (Mean Fluorescence intensity normalized with the number of cells in each image, p < 0.05 = *; p < 0.01 = **; p < 0.001 = ***; p < 0.0001 = ****)

Although variants of SLC10A7 cause a CDG, the function of SLC10A7 in Golgi glycosylation still remains unknown. To better characterize it, the migration profiles of two glycoproteins, TGN46 and LAMP2, were analyzed in patient-derived fibroblasts and isogenic SLC10A7 KO HAP1 cells complemented or not by the wild type form of SLC10A7 (Wt-SLC10A7) (A10 cells). Five patient fibroblasts (P1 to P5) were examined, with P1 and P2 (siblings-compound heterozygous) harboring c.722–16A > G and c.335G > A; p.[Gly112Asp] variants, and P3, P4, and P5 carrying c.221 T > C [p.Leu74Pro]; c.774-1G > A and c.514C > T [p.Gln172*] homozygous variants, respectively. An altered gel mobility was observed for both LAMP2 and TGN46 in SLC10A7-deficient patient fibroblasts indicating both *N*- and *O*- GalNAc glycosylation defects (Fig. 1A). These glycosylation defects observed on TGN46 were confirmed in control and P4 cells by a de-glycosylation assay using different glycosidases: *N*-glycanase (PNGase F), *O*-glycanase (endo-α-N-acetylgalactosaminidase), β(1,4)galactosidase, β-N-acetylglucosaminidase and sialidase A (Supplementary Fig. 1A and [14]). The result showed that while slightly N-glycosylated, the O-GalNAc glycosylation is predominant for TGN46. In order to prove the involvement of SLC10A7 in the observed glycosylation phenotype, we next investigated the glycosylation defect in SLC10A7 KO HAP1 cells. As before, the *N*- and *O*-GalNAc glycosylation status was assessed by analyzing the electrophoretic mobilities of LAMP2 and TGN46. Although no major differences were observed for LAMP2 (only a slight shift can be observed), a marked increased mobility for TGN46 was detected. Complementation by re-expression of wild-type SLC10A7 restored the TGN46-associated migration profile demonstrating the crucial involvement of SLC10A7 in maintaining the *O*- GalNAc glycosylation status of TGN46 (Fig. 1B). These results on LAMP2 and TGN46 were reminiscent of the ones observed in TMEM165 deficiency [14]. As previous work demonstrated the suppression of the TMEM165-associated glycosylation defects by using low Mn^2+^ concentrations [4, 15, 16], we wanted to investigate whether this was also the case in SLC10A7 deficiency. SLC10A7-deficient cells were thus treated with increasing Mn^2+^ concentrations and the electrophoretic mobilities of LAMP2 and TGN46 were reassessed. Our results show that, in patient fibroblasts and SLC10A7 KO HAP1 cells, low Mn^2+^ concentrations could not rescue the LAMP2 and TGN46 electrophoretic mobilities and hence a correct Golgi glycosylation (data not shown). However, and surprisingly, supplementation with high (supraphysiological, [17]) extracellular Mn^2+^ (500 µM for SLC10A7-CDG patient fibroblasts and 100 µM for SLC10A7 KO HAP1 cells) fully rescues the electrophoretic mobility of both LAMP2 and TGN46 in treated patient fibroblasts, and of TGN46 in treated SLC10A7 KO HAP1 cells (Fig. 1 A and C). Although the Mn^2+^ supplementation fully rescues the TGN46 migration profiles, the LAMP2 rescue appears to be incomplete and differs according to patients. Altogether, these results suggest that SLC10A7 deficiency is associated with severe Golgi glycosylation defects, mainly of the *O*-mucin type [18, 19], and that Mn^2+^ is capable of rescuing Golgi glycosylation, but not in the same concentration range as used for TMEM165 deficiency.

To characterize the *O*- GalNAc glycosylation defect, controls and two patient fibroblasts (P1 and P4) were treated or not with 500 µM MnCl_2_ and incubated with peracetylated benzyl-α-GalNAc (Bn-GalNAc) (Fig. 1D). When added to the culture media, this reporter is easily incorporated into the cells and deacetylated by cytosolic esterases. It then enters into the secretory pathway where it is modified by resident Golgi glycosyltransferases, and finally secreted into the media in which its glycosylation status can be analyzed by mass spectrometry [14, 20]. Obtained MALDI-QIT-TOF spectra are shown in Fig. 1D. In control fibroblasts, three main structures are found corresponding to bi-sialylated core 1 (m/z = 1316), mono and bi-sialylated core 2, the latter being the more abundant *O*-glycan structures (m/z = 1404 and 1765, respectively). Profiles of P1 and P4 patient cells are identical and differ quite significantly from the control. A total absence of core 2 structures is observed in both investigated patients compared to control, with the unique presence of mono and bi-sialylated core 1 structures (m/z = 955 and m/z = 1316, respectively). To confirm these results, the same experiment was performed in SLC10A7 KO HAP1 cells (Supplementary Fig. 1 B). In Wt cells, MALDI-QIT-TOF analysis revealed four main *O*-glycan structures corresponding to mono- and bi-sialylated core 1 (m/z = 955, 1316), as well as mono-sialylated and fucosylated core 2 (m/z = 1578). It has to be noted that the non-processed Bn-GalNAc (m/z = 390) strongly accumulates in SLC10A7 KO HAP1 cells compared to control cells, suggesting a galactosylation defect in these cells. This accumulation is concomitant to a reduction of the core 1-derived structures and the total absence of core 2 structures, as observed in the fibroblasts of patients. Complementation by reexpression of wild-type SLC10A7 not only fully restored the presence of core 2 *O*-glycan structures, but also strongly decreased the abundance of the non-processed Bn-GalNAc, demonstrating that the absence of SLC10A7 is fully responsible for the observed O-GalNAc glycosylation defect in SLC10A7 KO cells. Remarkably, while manganese supplementation rescued the electrophoretic mobility of TGN46, such treatment had no effect on Bn-GalNAc glycosylation status in patient cells. Moreover, supplementation with this MnCl_2_ concentration induces a severe glycosylation defect in control cells, similar to the one observed in patient fibroblasts treated or not with Mn^2+^ (Fig. 1 D). Altogether, these results demonstrate that manganese supplementation does not rescue the *O*-glycan structures in patient cells and induces a severe *O*- GalNAc glycosylation defect in control cells. The observed decreased core 2 biosynthesis in both cell types, as well as the increased abundance of non-processed Bn-GalNAc in SLC10A7 KO HAP1 cells, is evocative of core 1 galactosylation deficiency. To check this possibility, *Vicia villosa* lectin (VVL) coupled to fluorescein was used on non-permeabilized patient cells to evidence or not the presence of Tn antigen at the cell surface. VVL indeed preferentially recognizes α-terminal GalNAc residues linked with *O*-glycosidic bonds to serine or threonine in polypeptides. As shown in Fig. 1 E, the green signal associated to VVL-fluorescein was significantly higher in all SLC10A7-CDG patient fibroblasts compared to control cells, consistent with the presence of free terminal *O*-GalNAc residues exposed at the plasma membrane of SLC10A7-CDG patient cells. Altogether, these results demonstrate a function of SLC10A7 in Golgi *O*- GalNAc glycosylation, mainly in core 1 synthesis.

### SLC10A7 deficiency impacts the expression of both COSMC and C1GALT1 in patient fibroblasts

**Fig. 2:**
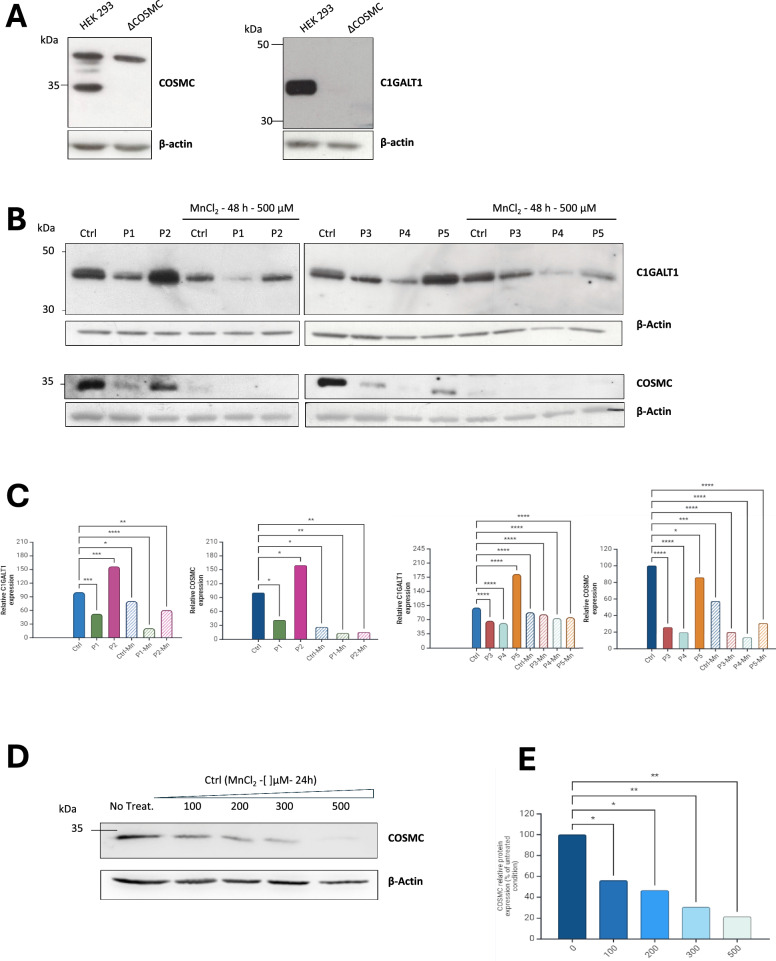
COSMC and C1GALT1 protein amounts are affected by SLC10A7 deficiency. **A** Western blot of COSMC and C1GALT1 in 293 T or COSMC KO HEK cells. **B** Western blot of C1GALT1 and COSMC in control and patient fibroblasts, treated or not with 500 µM MnCl_2_. **C** Quantification, normalized to actin and expressed in percentage of control signal. **D** Western blot of COSMC in control cells treated with increasing doses of MnCl_2_, from 0 to 500 µM. **E** COSMC signal quantification normalized to actin and expressed in percentage of untreated control (p < 0.05 = *; p < 0.01 = **; p < 0.001 = ***; p < 0.0001 = ****)

The *O*- GalNAc glycosylation defect caused by SLC10A7 deficiency appears to result from impaired synthesis of core 1 *O*-glycans, leading to subsequent core 2 defects. To investigate the underlying cellular mechanism, the abundance of the core 1 UDP-galactose:GalNAc-α-R β1,3-galactosyltransferase (C1GALT1) and COSMC, its molecular chaperone [21], were assayed by Western blot in patient fibroblasts treated or not with 500 µM MnCl_2_. COSMC KO HEK cells were used to check and confirm the specificity of the C1GALT1 and COSMC antibodies (Fig. 2A). The results obtained in patients, normalized to actin and expressed as a percentage of control, are shown in Fig. 2B and C. Three patients (P1, P3 and P4) exhibited a significant decrease in C1GALT1 levels, with a reduction of 40–50% compared to controls while two other patients (P2 and P5) had a higher expression level (nearly doubled) compared to controls. Intriguingly, a marked reduction in C1GAlT1 expression is observed in all cell lines including controls following MnCl_2_ supplementation. This suggests that SLC10A7 deficiency affects C1GALT1 levels and that supraphysiological MnCl_2_ concentrations strongly destabilize this glycosyltransferase. Given that C1GALT1 folding depends entirely on its ER chaperone COSMC, we also analyzed COSMC levels in the same samples. The results interestingly mirrored those observed for the C1GALT1: patients 2 and 5 showed a similar overexpression of COSMC, while other cell lines exhibited decreased COSMC expression. In the presence of high MnCl_2_ concentration, COSMC was strongly affected in all cell lines, including controls (Fig. 2B and C). To confirm this surprising sensitivity of COSMC to high MnCl_2_ doses, control cells were treated with increasing doses of MnCl_2_ to investigate the protein expression. The Western blot signals and their quantification normalized to actin are shown in Fig. 2 D and E. The results demonstrate that COSMC quantity is indeed decreased by MnCl_2_ in a dose-dependent manner, with concentrations ranging from 100 to 500 µM. As anticipated by the observed Mn induced COSMC decrease, C1GALT1 expression level was also found decreased followed increasing MnCl2 concentrations (data not shown). These findings indicate that (1) changes in C1GALT1 protein levels are correlated with COSMC levels, (2) SLC10A7 deficiency disrupts COSMC protein levels, and (3) high doses of MnCl_2_ fail to rescue glycosylation because they both decrease COSMC and C1GALT1 quantities in control cells, thus explaining the observed deleterious effects on Bn-GalNAc *O*-glycan structures.

### Supraphysiological MnCl_2_ concentrations increase GalNAc transfer onto proteins

**Fig. 3:**
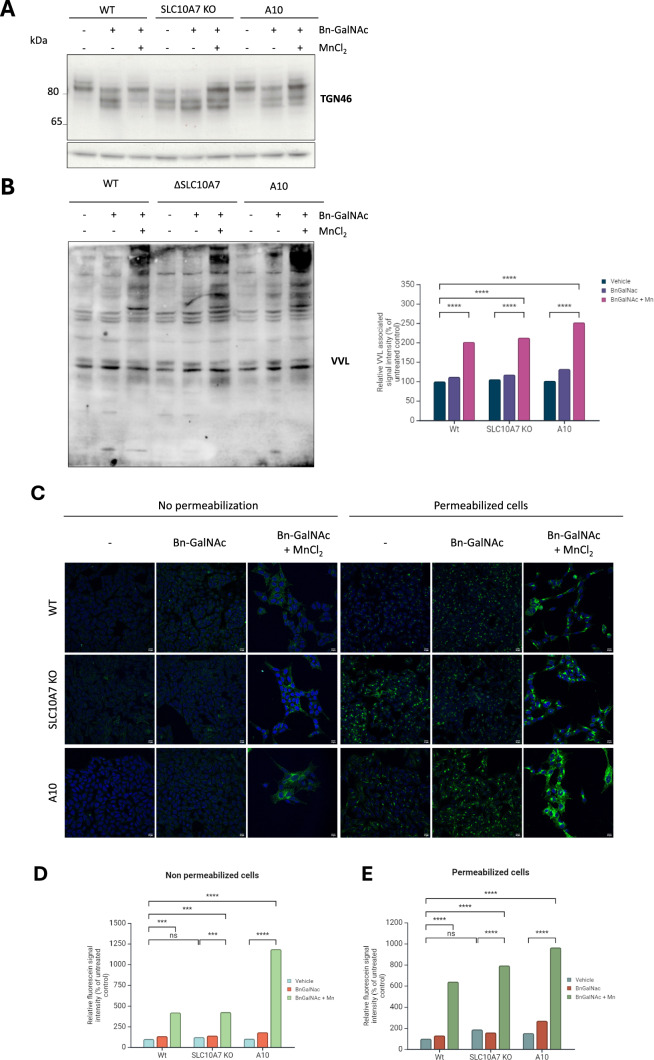
High MnCl_2_ doses increase unsubstituted GalNAc levels. **A** Western blot profile of TGN46 in Wt, SLC10A7 KO or A10 (SLC10A7 KO + Wt-SLC10A7) cells treated or not with 1 mM Bn-GalNAc, with or without 100 µM MnCl_2_ for 48 h. **B** VVL blot onto 10 µg of protein from Wt, SLC10A7 KO or A10 (SLC10A7 KO + Wt-SLC10A7) cells treated or not with 1 mM Bn-GalNAc with or without 100 µM MnCl_2_ for 48 h. **C** Quantification of VVL-associated signal. **D** Confocal images of VVL coupled with fluorescein in Wt, SLC10A7 KO or A10 (SLC10A7 KO + Wt-SLC10A7) cells treated or not with 1 mM Bn-GalNAC, with or without 100 µM MnCl_2_ for 48 h, and permeabilized with saponin for 10 min or not. **E** Quantification of the VVL-associated fluorescence signal (mean fluorescence intensity normalized to the number of cells per image) (p < 0.05 = *; p < 0.01 = **; p < 0.001 = ***; p < 0.0001 = ****)

Our results appear contradictory. How to explain, on the one hand, that MnCl_2_ supplementation affects both the stabilities of COSMC and C1GALT1 and, on the other hand, that such treatment rescues the electrophoretic mobility of TGN46? Since MnCl_2_ supplementation does not rescue the structure of the *O*-mucin type glycans, we hypothesized that MnCl_2_ supplementation could lead to a marked increase of GalNAc transfer onto *O*-glycosylation sites. To investigate this question, we took advantage of Bn-GalNAc to induce an *O*- GalNAc glycosylation defect by saturating C1GALT1 capacities. Cells were then treated with high doses of MnCl_2_ to see whether the transfer of GalNAc residues could be increased. We assessed this hypothesis by using the VVL lectin by blot and fluorescence staining. Wt, SLC10A7 KO or A10 HAP1 cells were thus treated with 1 mM Bn-GalNAc, with or without 100 µM MnCl_2_, and the migration profile of TGN46 was first assessed to see whether a TGN46 *O*- GalNAc glycosylation defect is induced by such treatment and rescued in the presence of manganese (Fig. 3 A). In Wt cells, the Bn-GalNAc treatment indeed led to a visible molecular shift of TGN46, indicating an *O*- GalNAc glycosylation defect. Interestingly, this shift is identical to the one observed in SLC10A7 KO cells without Bn-GalNAc treatment, thus confirming the galactosylation defect observed in SLC10A7 deficiency. As expected, the re-expression of wt-SLC10A7 in KO SLC10A7 cells led to the same glycosylation profile than that observed in control cells. Moreover, the addition of MnCl_2_ in all cell lines previously treated with Bn-GalNAc completely annihilates the Bn-GalNAc induced *O*- GalNAc glycosylation defect, as shown by the rescue of the TGN46 electrophoretic mobility. Interestingly, when VVL was used to assess the level of free *O*-GalNAc onto proteins, a marked increase (both by lectin blot and lectin fluorescence) was observed in all the samples treated with Bn-GalNAc and MnCl_2_ compared to the samples treated with Bn-GalNAc alone (Fig. 2 B, C and D). Taken together, these results demonstrate that the capacity of MnCl_2_ supplementation to rescue the electrophoretic mobility of TGN46 likely results from an increase of GalNAc transfer onto proteins.

### SLC10A7 deficiency impairs Ca^2+^ homeostasis in the secretory pathway

**Fig. 4:**
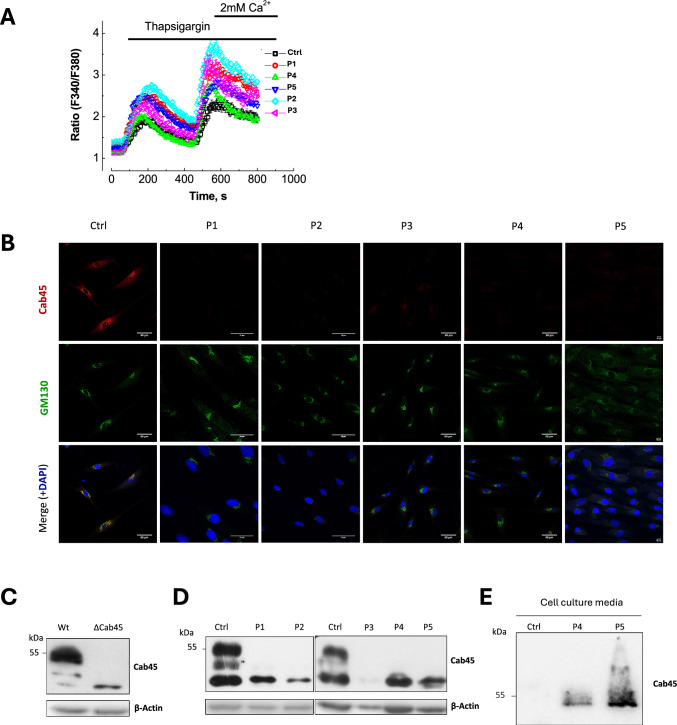
SLC10A7 deficiency induces a Ca.^2+^ increase within the secretory pathway and affects Cab45 localization. **A** Control and SLC10A7-deficient patient cells were loaded with Fura2/AM probe and subjected to calcium imaging experiment. Tg-induced SOCE was analyzed. **B** Confocal images of fixed control and SLC10A7-CDG patient fibroblasts (P1 to 5), immunostained with antibodies against Cab45 (red signal) and GM130, a Golgi marker (in green). DAPI blue was performed to mark the nucleus in blue. Merged images show an orange signal in case of a colocalization between GM130 and Cab45. **C** Western blot of Cab45 in Wt and Cab45 KO HAP1 cells. **D** Western blot of Cab45 in control and five SLC10A7-CDG patient fibroblasts. **D** Western blot of Cab45 on protein precipitates from cell culture media of control and two SLC10A7-CDG patient fibroblasts (P4 and P5). (p < 0.05 = *; p < 0.01 = **; p < 0.001 = ***; p < 0.0001 = ****)

Karakus et al*.* [12] showed that, in SLC10A7 KO HAP1 cells, Ca^2+^ entry via the SOC channels is increased, whereas the overexpression of SLC10A7 inhibits the SOC-dependent Ca^2+^ entry, suggesting that SLC10A7 expression negatively correlates with SOC-dependent Ca^2+^ entry. To monitor SOCE (Store Operated Calcium Entry, or the entry of Calcium through the plasma membrane channel Orai1 following the ER stocks depletion) in our SLC10A7-deficient patient fibroblasts, Ca^2+^ influx in Fura-2-loaded cells was monitored after depleting ER Ca^2+^ stores with 10 mM thapsigargin (Tg) in Ca^2+^-free solution followed by incubation with a solution containing 2 mM Ca^2+^ [22]. After Tg treatment, the observed Ca^2+^ release was found increased in all SLC10A7-deficient patient cell lines, compared to control cells, thus showing a higher ER Ca^2+^ content in patient cells and supporting the increase in SOCE activity in the absence of functional SLC10A7. This was further confirmed by the addition of 2 mM Ca^2+^ that led to a 2- fold increase in Ca^2+^ influx rate in patient cells compared to control cells. Altogether, these results confirmed a general impairment of Ca^2+^ homeostasis in SLC10A7-deficient cells, with an accumulation of Ca^2+^ in organelles, likely the ER, and an increased Ca^2+^ entry via SOC channels. We next explored whether the SLC10A7 defect could also affect Golgi Ca^2+^ homeostasis. As Ca^2+^ measurement in the Golgi is much more complex than in the ER, we investigated the expression and subcellular localization of Cab45, a *trans*-Golgi soluble protein whose oligomerization at the TGN level depends on Golgi Ca^2+^ homeostasis. Intriguingly, while Cab45 (red) fully colocalizes with the Golgi marker GM130 (green) in control fibroblasts, no signal was detected in the five investigated SLC10A7-CDG patient fibroblasts (Fig. 4B). This complete loss of Cab45 was confirmed by Western blot in all SLC10A7-CDG patient fibroblasts where, unlike in control cells, a total absence of target protein band can be seen. The antibody specificity was assessed thanks to Cab45 KO HAP1 cells (Fig. 4C and D). The use of lysosomal inhibitors did not rescue the abundance of Cab45 in patient cells (data not shown). As Cab45 is required for *trans*-Golgi network Ca^2+^ homeostasis and sorting of cargos that are destined for secretion, we considered that the observed lack of Cab45 could result from its secretion. To check this possibility, the abundance of Cab45 was assessed in the culture medium of two patient cells compared to controls by Western blot after precipitation of the proteins from the cell culture medium. The result depicted in Fig. 4D shows that Cab45 is absent from the culture supernatant of control cells while it is detected in the culture supernatant from both P4 and P5 patients. Altogether, these findings suggest that (1) SLC10A7 is a key protein in controlling Ca^2+^ in the secretory pathway and (2) SLC10A7’s function is required for Cab45 Golgi localization.

## Discussion

Although SLC10A7 deficiency leads to a CDG associated with strong Golgi glycosylation defects, the function of SLC10A7 and its role in glycosylation are unknown [5, 6, 14]. As for TMEM165, the SLC10A7 structure teaches us that it is not directly involved in glycosylation as it is neither a Golgi glycosylation enzyme, nor a XDP-sugar transporter [10]. In this study, we show that SLC10A7 deficiency leads to altered electrophoretic migration profiles for TGN46 and LAMP2 indicative for both *N*- and *O*- GalNAc glycosylation defects. The use of Bn-GalNAc confirmed the *O*- GalNAc glycosylation defects in SLC10A7-deficient cells that exhibited a complete loss of core 2 *O*-glycans along with a decrease in core 1 *O*-glycans. Several hypotheses can be formulated. Although we can’t completely exclude a direct defect in GlcNAc transfer, our results favor a defect in core 1 *O*-glycan synthesis [19, 23]. Core 1 *O*-glycan synthesis is known to be controlled by the activity of core 1 GalT (C1GALT1) which requires the activity of its specific ER molecular chaperone COSMC to fold and be expressed properly [21, 24]. Therefore, defects in COSMC have been associated to C1GALT1 defects resulting in the presence of Tn antigen, the simplest *O*-glycan found onto proteins (GalNAcα1-O-Ser/Thr). The cellular levels of this Tn antigen are associated with cancer aggressiveness, poor prognosis and metastasis [25, 26]. Amongst the factors that regulate the *O*-GalNAc glycosylation, the subcellular localization of the ppGalNAc-Ts and their recycling strongly affect the quantity of Tn antigen, as well as factors that affect the elongation of core O-glycans such as COSMC and/or the C1GALT1 [27, 28]. The understanding of this regulation is fundamental with strong medical implications. Our results highlight that SLC10A7 is an unexpected key Golgi transmembrane protein in the regulation of this pathway, controlling the abundance of COSMC and C1GALT1. The molecular mechanisms by which a lack of SLC10A7 affects COSMC expression are not known but could result from a general ER metal homeostasis impairment. Previous studies by Cummings et al*.* have indeed shown that the oligomerization and activity of COSMC, would be dependent on ER zinc homeostasis [29]. This has recently been confirmed in ZIP9 deficiency that induces a marked increased expression of truncated *O*-glycans resulting from Zn^2+^ depletion in the secretory pathway [30]. Our results demonstrating that supraphysiological manganese concentration strongly affects COSMC abundance and in turn C1GALT1, are somehow in line with the previously observed Zn^2+^ metal dependency of COSMC. Although not fully solved yet, this could result from a mismetallation of COSMC by Mn^2+^ that would then dislodge Zn^2+^. Intriguingly, an ER Ca^2+^ homeostasis impairment could also result in this mismetallation phenomenon thus leading to the observed differential COSMC expression in SLC10A7-CDG patients. Indeed, our results highlight that SLC10A7 deficiency leads to a global impairment of Ca^2+^ homeostasis in the secretory pathway, confirming the previous results from Karakus and collaborators in HAP1 cells. They indeed found that SLC10A7 is a negative regulator of SOC-dependent Ca^2+^ entry in cells [12, 13, 31]. In line with their findings, our results demonstrate that the lack of SLC10A7 in patient cells leads to an overall accumulation of Ca^2+^ in the secretory pathway resulting from an overactivated Ca^2+^ entry via SOC channels. The ER Ca^2+^ content, assessed by the use of thapsigargin, increasing cytosolic calcium levels through ER calcium stores release, was found twice as high as in controls. Interestingly, this ER accumulation echoes in the Golgi. This was assessed by following the expression and localization status of Cab45, a *trans*-Golgi soluble protein, that oligomerizes in the presence of Ca^2+^ [32, 33]. Surprisingly, while Cab45 is normally found in the Golgi of control fibroblasts, a total absence of the protein was observed in all the investigated SLC10A7-CDG patient cells. Our findings highlight that this absence is correlated with a secretion of Cab45, as demonstrated by its presence in the culture media of two SLC10A7-deficient patients. Besides the fact that this extracellular localization could be considered as a marker of SLC10A7 deficiency, Cab45 could also be used as an elegant sensor of Golgi Ca^2+^ homeostasis impairment in different pathophysiological contexts affecting Golgi.

Although our results unambiguously demonstrate that Mn^2+^ excess is detrimental for the *O*- GalNAc glycosylation pathway in control and SLC10A7-deficient cells, manganese supplementation mysteriously rescued the TGN46 molecular weight. How to reconcile these two opposite effects? The use of VVL staining in HAP1 cells treated with both Bn-GalNAc, that induces an *O*- GalNAc glycosylation defect, and manganese gave us some clues on the origin of this TGN46 molecular weight rescue. Actually, all our results converge on an artefact of the manganese supplementation that would lead to a marked increase of GalNAc transfer onto proteins. The GalNAc transfer from UDP-GalNAc onto Ser or Thr residues of proteins is initiated by a family of polypeptide GalNAc-transferases (ppGalNAcTs; E.C. 2.4.1.41) that are conserved across metazoan with 20 different human isoenzymes identified so far, and whose regulation is essential for the initiation of *O*-GalNAc glycosylation [34]. These enzymes are Golgi type II transmembrane proteins having the singularity to possess two essential domains: the luminally-oriented catalytic one which is manganese dependent, and a lectin domain at the C-terminal part of the protein connected by a flexible linker to the catalytic domain. This lectin domain that binds to GalNAc residues on glycopeptides is of fundamental importance in enhancing the transfer of other GalNAc residues by most GalNAc-Ts toward an acceptor site 7 to 11 amino acids away [35, 36]. The metal dependency of this lectin domain, which resembles that of ricin B-type lectins, is not yet explained but could well be dependent on Ca^2+^[37]. If this holds true, the function of ppGalNAc-Ts would then be tightly regulated by the Ca^2+^/ Mn^2+^ homeostasis within the secretory pathway. In SLC10A7 deficiency where an increased Ca^2+^ homeostasis is observed, the binding of this lectin domain could be enhanced, thus preventing the C1GALT1 to act. In case of manganese supplementation, the ppGalNAc-Ts enzymatic activities could be increased, leading to the transfer of higher amounts of GalNAc residues onto proteins. We believe that the impact of Ca^2+^/ Mn^2+^ homeostasis on the regulation of this process constitutes a fascinating challenge for the future.

Altogether, our results show that SLC10A7 is crucial in tightly regulating Ca^2+^ homeostasis in the entire secretory pathway, required for COSMC quantity and Cab45 proper TGN localization. It could well be that Zn-dependent COSMC functioning/ quantity could either be disrupted by excess of Mn^2+^ (in case of supraphysiological supplementation) and Ca^2+^ (in case of SLC10A7 deficiency) in the ER, likely by mismetallation of the molecular chaperone. This would make COSMC as a highly sensitive protein to ER metal homeostasis changes. This work underlines that the initiation/ extension of *O*- GalNAc glycosylation is tightly regulated by ER/ Golgi metal homeostasis in which SLC10A7 is a key player. This work reinforces the fundamental importance of metals homeostasis in glycosylation [38, 39]

## Supplementary Information

Below is the link to the electronic supplementary material.

Supplementary file 1 (DOCX 129 KB)

## Data Availability

The data are available upon request from the corresponding authors.

## Abbreviations

CDG: Congenital disorder of glycosylation
ER: Endoplasmic reticulum
SLC: Solute carrier
TGN: *Trans*-Golgi network
Cab45: Calcium-binding protein of 45 kDa
PBS: Phosphate-buffered Saline
Ac_3_ Bn-GalNAc: Peracetylated-Benzyl-O-*N*-acetylgalactosamine
Tg: Thapsigargin
GalNAc: *N*-Acetylgalactosamine
GlcNAc: *N*-Acetylglucosamine
Fuc: Fucose
Sia: Sialic acid
Man: Mannose
Gal: Galactose
SOCE: Store-operated calcium entry
