# Supplementary material for: SLC10A7 regulates O-GalNAc glycosylation and Ca2+ homeostasis in the secretory pathway: insights into SLC10A7-CDG
Source: Cell Mol Life Sci. 2025 Jan 8;82(1):40. doi: 10.1007/s00018-024-05551-2 (PMC11711720; doi:10.1007/s00018-024-05551-2)
Supplement: Supplementary file 1 — (DOCX 129 KB) [file 18_2024_5551_MOESM1_ESM.docx]

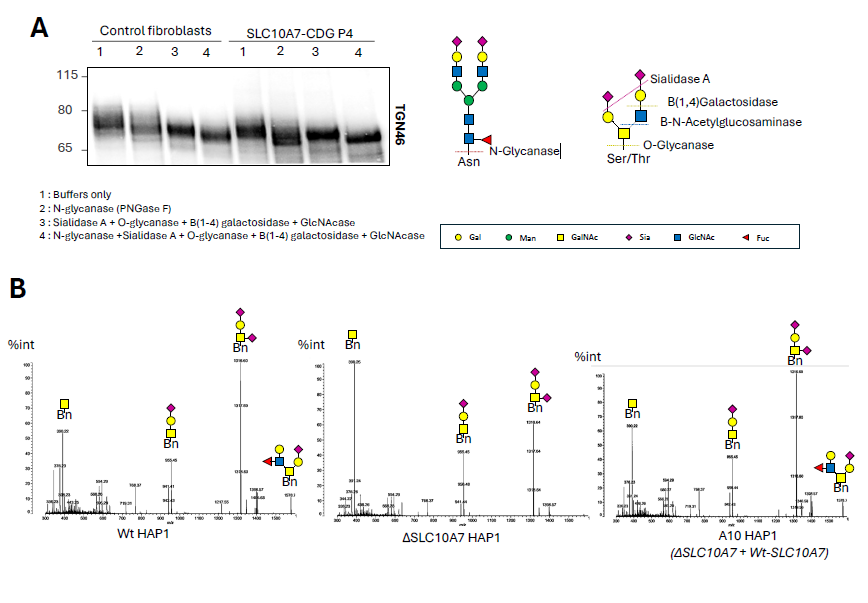


**Supplementary Figure 1 : Characterization of SLC10A7-induced glycosylation defect. A.** Deglycosylation assay of protein lysates from control and P4 SLC10A7-CDG patient fibroblasts. Hundred µg of lysates were denaturated and incubated with the deglycosylation enzymes indicated in the figure, for 3 hours at 37°C. Samples were then submitted to a TGN46 Western blot. The enzymes and the cleavage they catalyze are indicated on the right, and the symbols depicting the monosaccharides are found right below. **B.** MALDI-QIT-TOF spectra of Bn-GalNAc glycosylation status in Wt, SLC10A7 KO or A10 (SLC10A7 KO + Wt-SLC10A7) cells, treated with Ac_3_-Bn-GalNAc for 72 hours.
